# Case report: Endovascular stent-graft repair of aortic penetrating trauma, literature review, and case report

**DOI:** 10.3389/fsurg.2023.1264558

**Published:** 2023-10-11

**Authors:** Juan Gabriel Bayona, Carlos Eduardo Rey Chaves, Oscar Geovanny Hernández Rodríguez, Vladimir Barón, Ernesto Fajardo, Eduardo Posada

**Affiliations:** ^1^ Cirujano General, Pontificia Universidad Javeriana, Bogotá, Colombia; ^2^ Estudiante de Posgrado Cirugía General, Pontificia Universidad Javeriana, Facultad de Medicina, Bogotá, Colombia; ^3^ Cirugía Vascular Periférica, Pontificia Universidad Javeriana, Facultad de Medicina, Hospital Universitario San Ignacio, Bogotá, Colombia

**Keywords:** aortic pseudoaneurysm, penetrating trauma, endovascular repair, vascular injury, outcomes

## Abstract

Penetrating aortic injuries are infrequent. Its incidence is unknown because most patients die of hemorrhage even before they receive adequate treatment. Aortic wounds generally require conventional thoracotomy/laparotomy repair and are related to high mortality rates. Recently with the advent of endovascular techniques, most authors prefer endovascular management when feasible due to better (still poor) outcomes. The short- and mid-term results of immediate endovascular repair of traumatic aortic injuries are promising, especially when compared with open surgical treatment, indicating that endovascular therapy is preferable in patients with multi-trauma and traumatic ruptures of the thoracic aorta. Here we present the diagnosis and treatment of a 30 years-old male patient with multiple traumatic stab wounds, including anterior aortic laceration with a grade II aortic lesion successfully managed with an endovascular stent graft.

## Introduction

Penetrating aortic injuries are uncommon. Its incidence is unknown because most patients die even before they receive adequate treatment due to massive hemorrhage (1, 2). Aortic wounds generally require a conventional open surgical approach (thoracotomy or laparotomy) this open approach has been associated, among many other complications, with a 28% mortality rate and 16% paraplegia rate (3, 4). Endovascular aortic repair (EVAR) is a rapidly developing technique that involves placing an endovascular stent graft in the abdominal or thoracic aorta, avoiding the morbidity of open surgery, cardiopulmonary bypass, and aortic cross-clamping (5–7). However, despite recent improvements in resuscitation and emergency operative techniques, penetrating aortic trauma outcomes are still associated with high mortality (5–11). Therefore, we present the diagnosis and treatment of a patient with multiple traumatic stab wounds, including anterior aortic laceration with a grade II aortic lesion (10) successfully managed with an endovascular stent graft Informed consent has been obtained from the patient for publication of the case report and accompanying images.

## Case report

**Figure 1:**
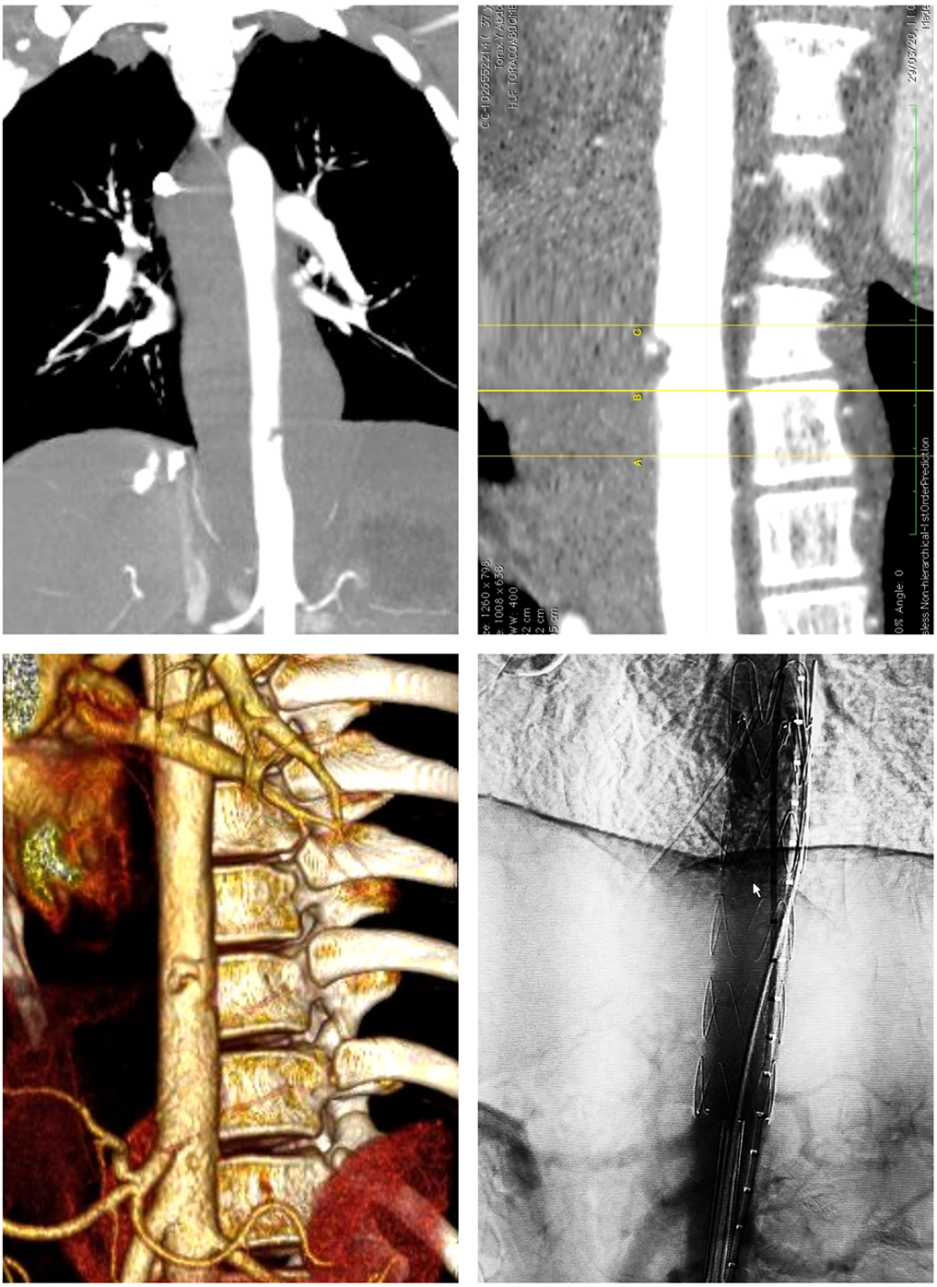
Angiogram showing the location of the aortic lesion, the measured distance from the aortic transection to celiac trunk and the aortic 3d reconstruction.

After ethical and institutional approval, previous informed consent was filled, following SCARE guidelines (11). We present a 30-year-old man who was found unconscious in a public pathway and transported to the emergency room with multiple thoracic and abdominal stab wounds. At the emergency room, patient presented with tachycardia (Heart rate 116), with an arterial pressure of 90/60 mmHg; initial management was performed with 1l of Ringer lactate. Preoperative blood transfusion was not required. Immediately after initial treatment due to the localization of external wounds and chest x-ray images, the vascular surgery team was called for evaluation. Four knife wounds were found on physical examination. A left supraclavicular entry orifice in zone 1 of the neck was evident without an exit wound, a right paramedian subscapular superficial wound was also evident, a left subxiphoid penetrating wound, and another penetrating wound on the right flank. The patient was hemodynamically unstable at initial hospital admission but responded to initial reanimation strategies with crystalloids. Based on the suspicion of large vessel trauma, Angio tomographic images were ordered and reviewed under multiplanar and 3D analysis (Figure 1). A descendent thoracic periaortic hematoma compromising the whole circumference with a flap-type injury at the diaphragm level was noted (Figure 1). This hematoma involved Ishimaru zones 3 and 4 and started 140 mm from the origin of the left subclavian artery and ended distally at 38 mm from the origin of the celiac trunk. There was no evidence of injuries to the abdominal aorta or other major vascular structures. These findings were later confirmed by contrast aortography.

**Figure 2:**
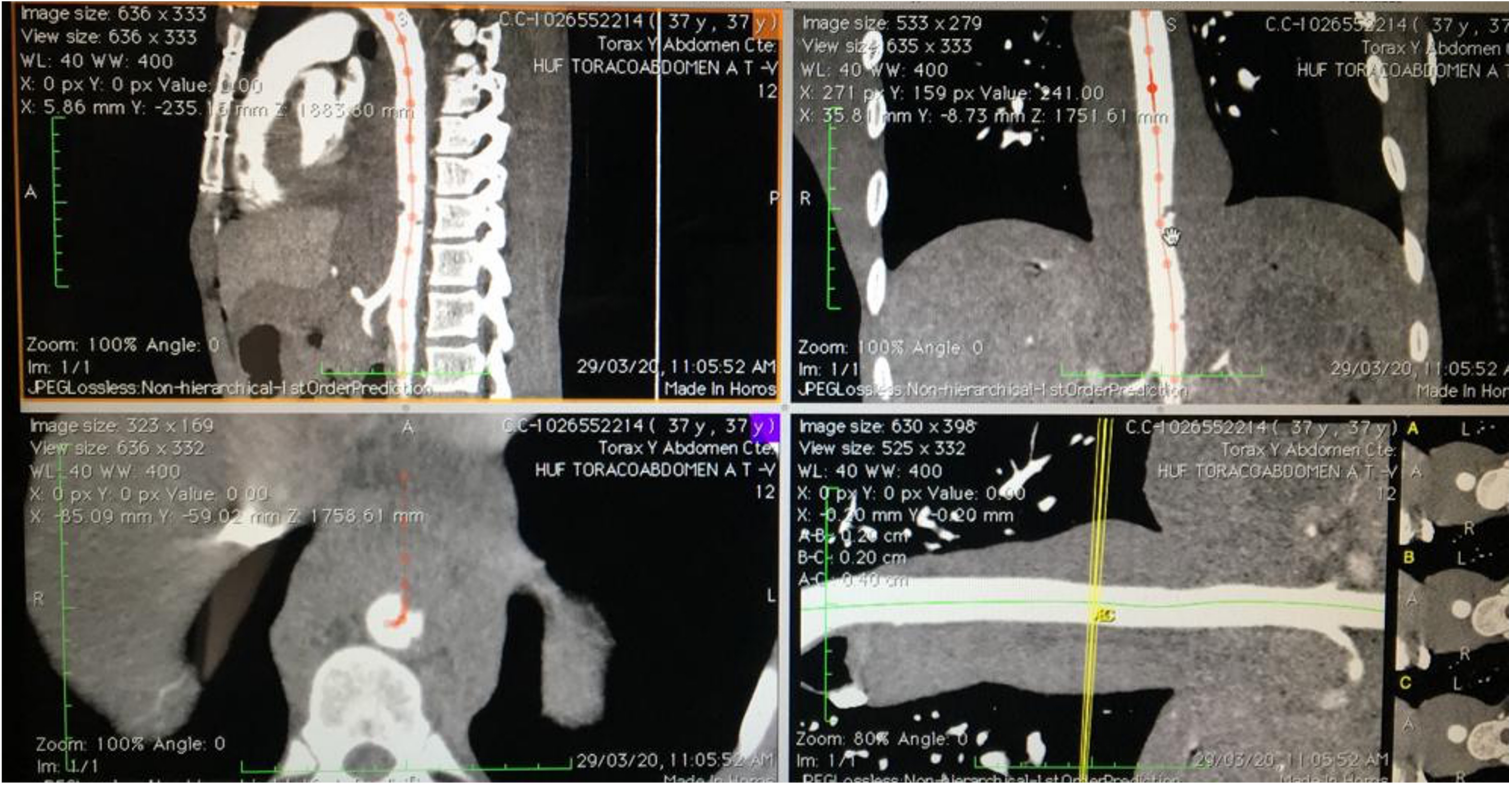
CT preoperative planification, measurement of the aorta.

**Figure 3:**
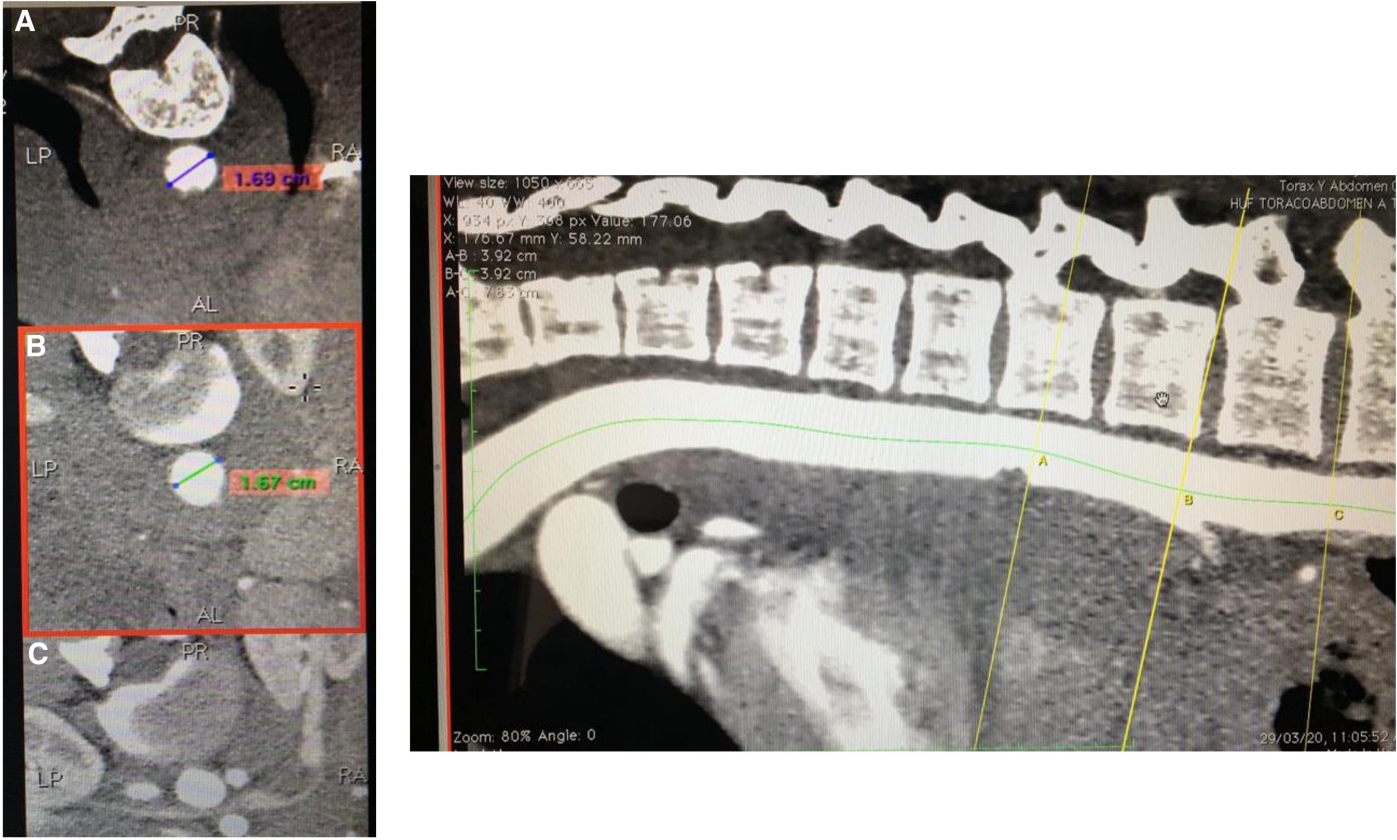
CT angiography measurement of the aorta, distance to the ostium of the celiac trunk.

Emergency endovascular stenting was the best treatment option considering the area compromised because of its proximity to the crura (accessing the supradiaphragmatic aorta is likely to be challenging via open techniques), also due to the least invasive approach given the patient's young age, stable retroperitoneal and thoracic supporting connective tissue/hematoma, and a relatively low, stable blood pressure despite his critical state and the imminent risk of rupture and death. Preoperative measurement of the aorta, with an oversize of 20% was performed to select the endograft size (Figures 2, 3).

Under local anesthesia, both femoral arteries were percutaneously punctured guided by ultrasound. After heparinization, the right and then left common femoral arteries were punctured, an 8 Fr right Pinnacle sheath (Terumo, Somerset NJ). Two ProGlide arterial closure systems were placed on each arterial access.

A hydrophilic 0.035 × 260 mm *GlideWire* (Terumo, Somerset NJ) wire was advanced into the aortic arch via the right femoral Pinnacle sheath, followed by a multipurpose catheter. The hydrophilic wire was posteriorly exchanged for a high support 0.035 × 260 mm *Lunderquist* (cook Medical INC., Bloomington, In) wire. A *Lunderquist* guidewire 0.035 × 260 mm (cook Medical INC., Bloomington, In) was advanced into the aortic arch via the left femoral Pinnacle sheath, followed by a pigtail centimeter marked catheter. The celiac trunk was marked. The right femoral Pinnacle sheath was removed, and the endoprosthesis was inserted (cook zenith® Alpha 16 fr) (cook Medical INC., Bloomington, In). The aortic stent was deployed, preserving the celiac trunk (Figure 1). After confirmation of adequate celiac trunk flow, the free flow was released. A completion aortogram via the left pigtail catheter demonstrated the lesion's satisfactory exclusion, showing a well-placed endoprosthesis in the abdominal aorta with no evidence of active bleeding or contrast leakage and adequate patency of the celiac trunk (Figure 1). The catheters were removed, the femoral punctures were repaired using Proglide systems, and the groins closed.

Immediately after the repair of the aortic lesion, a diagnostic laparotomy was performed under general anesthesia with better vital signs. Findings of this second procedure were 2,500 cc of hemoperitoneum, a grade II injury of segment II of the liver, a grade I injury of the upper pole of the spleen, a pinpoint injury of proximal ileum at 130 cms from the ligament of *Treitz*, and a grade I injury of ascending colon. Blood was drained, lesions to the colon and ileum were managed with primary suture repair and lesions to the liver and spleen with manual compression. After the procedure, there were no visible bleeding spots.

The patient had an adequate recovery without complications. The patient's discharge was made after six days of medical observation. At 6-weeks follow up the patient had no device, access site, or systemic complications. Contrast Tomography (CT) follow-up is planned at regular intervals.

## Discussion

Aortic penetrating trauma is uncommon and usually a lethal condition. It occurs in less than 2% of all patients with penetrating trauma and has an approximate mortality of 80%, reaching 87% when a gunshot is the mechanism of trauma (9, 10, 12). In the United States, blunt trauma is the leading cause of aortic injury, for which endovascular repair is a well-defined and increasing practice with evidence of decreased mortality and post-operatory complications (13) In contrast, penetrating vascular trauma due to gunshots or stab wounds in developing countries such as Colombia is still more prevalent than blunt trauma (14, 15) Nevertheless, the experience with penetrating aortic trauma is limited because of its high mortality.

Now, the literature concerning cases of successful endovascular therapy is scarce. Nevertheless, according to the actual evidence endovascular approach should be the first option for treatment for this traumatic condition according to Rimbau et al. (16) in the European Society of Vascular Surgery guidelines (16). Multiple studies compare the open approach vs. Thoracic endovascular aortic repair (TEVAR) in traumatic conditions and show a reduction in mortality (9.7% vs. 27.7%) with statistically significant value, and a lesser rate of neurologic complications such as paralysis and stroke rate (0.4% vs. 2.9% and 0.4% vs. 2.3) (6, 17, 18). As well, Hoffer et al. Hoffer et al. found a reduction in mortality from 20.2% to 8.4% (*p* = 0.001, *n* = 638) and a reduction in Paralysis from 5.7% to 0.83% (*p* = 0.001, *n* = 638) (18) These results are consistent with the ones reviewed by Karmy–Jones et al. in 2011 (6). For that reason, actual recommendations suggest that for patients with suitable anatomy endovascular approach should be considered first than the open approach (10, 16–19).

Penetrating aortic trauma frequently affects adjacent tissues and organs such as the esophagus, diaphragm, and heart, also, in most cases patients die before reaching hospital facilities (20). In a large proportion of cases, the patients enter the emergency room with hemodynamic instability; in these conditions, any imaging method should be performed, and the surgical approach should not be delayed (16, 20). If the rupture has a contained hematoma, as reported in this patient, endovascular repair may be deferred until the associated life-threatening trauma is treated. In this case, the priority was the aortic wound and the evaluation of additional abdominal or thoracic diaphragm wounds was postponed. The short and mid-term results of immediate endovascular repair of traumatic aortic injuries are promising, especially when compared with open surgical treatment, indicating that endovascular therapy is preferable in patients with multi-trauma and traumatic ruptures of the thoracic aorta (6, 8, 13).

There is scarce literature regarding a long-term follow-up for patients treated with the endovascular approach, nevertheless, according to the data published by Cheng et al. (21), there is a lesser rate of reintervention after 1 and 5 years for patients treated with the TEVAR approach compared with open techniques (0% vs. 2.6%), as well, neurologic complications were lesser in TEVAR approach (21) however, further prospective studies are needed in order to evaluate long-term outcomes for this patients.

This case exhibits a patient hemodynamically stable after initial reanimation with crystalloids with a penetrating aortic injury, an intramural hematoma, and active bleeding successfully treated with TEVAR. A less invasive procedure was made without the necessity of aortic clamping and the morbidity associated with a thoracotomy or a sternotomy.

## Conclusions

In conclusion, in patients with multi-trauma and traumatic rupture of the thoracic aorta, performing endovascular therapy is promising for the short- and mid-term results compared with conventional thoracotomy repair. With the increasing use by vascular surgeons of endovascular techniques for traumatic and nontraumatic aortic urgencies, these interventions will likely play an essential role in the future. However, most penetrating aortic trauma is presented in young patients with substantial life expectancy. Subsequent clinical studies evaluating the use of long-term aortic endograft must be performed.

## Data Availability

The raw data supporting the conclusions of this article will be made available by the authors, without undue reservation.

## References

[1] Parmley LF Mattingly TW Manion WC. Penetrating wounds of the heart and aorta. Circulation. (1958) 17(5):953–73. 10.1161/01.CIR.17.5.953 13537286

[2] Demetriades D Theodorou D Murray J Asensio JA Cornwell EE 3rd Velmahos G Mortality and prognostic factors in penetrating injuries of the aorta. J Trauma Acute Care Surg. (1996) 40(5). 10.1097/00005373-199605000-00013 8614076

[3] Chiba K Abe H Kitanaka Y Miyairi T Makuuchi H. Conventional surgical repair of traumatic rupture of the thoracic aorta. Gen Thorac Cardiovasc Surg. (2014) 62(12):713–9. 10.1007/s11748-014-0422-x 24902929 PMC4254169

[4] Canaud L Alric P Branchereau P Joyeux F Hireche K Berthet JP Open versus endovascular repair for patients with acute traumatic rupture of the thoracic aorta. J Thorac Cardiovasc Surg. (2011) 142(5):1032–7. 10.1016/j.jtcvs.2010.11.051 21397264

[5] Urgnani F Lerut P Da Rocha M Adriani D Leon F Riambau V. Endovascular treatment of acute traumatic thoracic aortic injuries: a retrospective analysis of 20 cases. J Thorac Cardiovasc Surg. (2009) 138(5):1129–38. 10.1016/j.jtcvs.2008.10.057 19660375

[6] Karmy-Jones R Ferrigno L Teso D Long WB 3rd Shackford S. Endovascular repair compared with operative repair of traumatic rupture of the thoracic aorta: a nonsystematic review and a plea for trauma-specific reporting guidelines. J Trauma Acute Care Surg. (2011) 71(4). 10.1097/TA.0b013e3182288783 21986746

[7] Kolbeck KJ Kaufman JA. Endovascular stent grafts in urgent blunt and penetrating thoracic aortic trauma. Semin Intervent Radiol. (2011) 28(1):98–106. 10.1055/s-0031-1273944 22379280 PMC3140249

[8] Neuhauser B Czermak B Jaschke W Waldenberger P Fraedrich G Perkmann R. Stent-graft repair for acute traumatic thoracic aortic rupture. Am Surg. (2004) 70(12):1039–44. 10.1177/000313480407001202 15663041

[9] Orlas CP Herrera-Escobar JP Zogg CK Serna JJ Meléndez JJ Gómez A Chest trauma outcomes: public versus private level I trauma centers. World J Surg. (2020) 44(6):1824–34. 10.1007/s00268-020-05400-w 31993723 PMC7380545

[10] Lee WA Matsumura JS Mitchell RS Farber MA Greenberg RK Azizzadeh A Endovascular repair of traumatic thoracic aortic injury: clinical practice guidelines of the society for vascular surgery. J Vasc Surg. (2011) 53(1):187–92. 10.1016/j.jvs.2010.08.027 20974523

[11] Agha RA Franchi T Sohrabi C Mathew G Kerwan A, SCARE Group. The SCARE 2020 guideline: updating consensus surgical CAse REport (SCARE) guidelines. Int J Surg. (2020) 84:226–30. 10.1016/j.ijsu.2020.10.034 33181358

[12] Eckert KL. Penetrating and blunt abdominal trauma. Crit Care Nurs Q. (2005) 28(1). 10.1097/00002727-200501000-00005 15732423

[13] Xenos ES Abedi NN Davenport DL Minion DJ Hamdallah O Sorial EE Meta-analysis of endovascular vs open repair for traumatic descending thoracic aortic rupture. J Vasc Surg. (2008) 48(5):1343–51. 10.1016/j.jvs.2008.04.060 18632242

[14] Ordoñez CA Manzano-Nunez R Naranjo MP Casualties of peace: an analysis of casualties admitted to the intensive care unit during the negotiation of the comprehensive Colombian process of peace. World J Emerg Surg. (2018) 13(1):2. 10.1186/s13017-017-0161-2 29371879 PMC5769432

[15] Svensson LG Kouchoukos NT Miller DC Bavaria JE Coselli JS Curi MA Expert consensus document on the treatment of descending thoracic aortic disease using endovascular stent-grafts. Ann Thorac Surg. (2008) 85(1):S1–S41. 10.1016/j.athoracsur.2007.10.099 18083364

[16] Riambau V Böckler D Brunkwall J Cao P Chiesa R Coppi G Editor’s choice management of descending thoracic aorta diseases: clinical practice guidelines of the European society for vascular surgery (ESVS). Eur J Vasc Endovasc Surg. (2017) 53(1):4–52. 10.1016/j.ejvs.2016.06.005 28081802

[17] Steuer J Björck M Sonesson B Resch T Dias N Hultgren R Editor’s choice – durability of endovascular repair in blunt traumatic thoracic aortic injury: long-term outcome from four tertiary referral centers. Eur J Vasc Endovasc Surg. (2015) 50(4):460–5. 10.1016/j.ejvs.2015.05.012 26143100

[18] Hoffer EK Forauer AR Silas AM Gemery JM. Endovascular stent-graft or open surgical repair for blunt thoracic aortic trauma: systematic review. J Vasc Interv Radiol. (2008) 19(8):1153–64. 10.1016/j.jvir.2008.05.012 18656007

[19] Ding X Jiang J Su Q Hu S. Endovascular stent graft repair of a penetrating aortic injury. Ann Thorac Surg. (2010) 90(2):632–4. 10.1016/j.athoracsur.2010.01.079 20667365

[20] Slocum C Chiba H Emigh B Tam B Schellenberg M Inaba K Nationwide analysis of penetrating thoracic aortic injury: injury patterns, management, and outcomes. J Surg Res. (2023) 284:290–5. 10.1016/j.jss.2022.11.077 36621259

[21] Cheng YT Cheng CT Wang SY Wu VC Chu PH Chou AH Long-term outcomes of endovascular and open repair for traumatic thoracic aortic injury. JAMA Netw Open. (2019) 2(2):e187861. 10.1001/jamanetworkopen.2018.7861 30735232 PMC6484615

